# The effect of parathyroidectomy compared to non-surgical surveillance on kidney function in primary hyperparathyroidism: a nationwide historic cohort study

**DOI:** 10.1186/s12902-021-00918-z

**Published:** 2022-01-06

**Authors:** Josephine Matzen, Lise Sofie Bislev, Tanja Sikjær, Lars Rolighed, Mette Friberg Hitz, Pia Eiken, Anne Pernille Hermann, Jens-Erik Beck Jensen, Bo Abrahamsen, Lars Rejnmark

**Affiliations:** 1 Department of Endocrinology and Internal Medicine, Aarhus University Hospital, Aarhus, Denmark; 2 Department of Otorhinolaryngology, Aarhus University Hospital, Aarhus, Denmark; 3 Department of Medical Endocrinology, Zealand University Hospital Køge, Køge, Denmark; 4 Institute of Clinical Medicine, University of Copenhagen, Copenhagen, Denmark; 5 Department of Endocrinology, Bispebjerg, Copenhagen University Hospital, Copenhagen, Denmark; 6 Department of Endocrinology, Odense University Hospital, Odense, Denmark; 7 Department of Endocrinology, Copenhagen University Hospital Hvidovre, Hvidovre, Denmark; 8 Department of Clinical Research, University of Southern Denmark and Odense University Hospital, Odense C, Denmark; 9 Holbæk Hospital, Department of Medicine, Holbæk, Denmark; 10 Institute of Clinical Medicine, Aarhus University, Aarhus, Denmark

**Keywords:** Primary hyperparathyroidism, Parathyroid glands, Parathyroidectomy, Renal function, Kidney function, Parathyroid hormone

## Abstract

**Background:**

Patients with primary hyperparathyroidism (pHPT) and impaired kidney function (estimated glomerular filtration rate (eGFR) < 60 mL/min) are offered parathyroidectomy (PTX) to protect them from further complications. Surprisingly, two recent uncontrolled cohort studies have suggested a further decrease in kidney function following PTX. We aimed to examine the effects of PTX compared to non-surgical surveillance on kidney function in pHPT patients.

**Methods:**

Historic cohort study. From the Danish National Patient Registry (NPR) and major medical biochemistry laboratories in Denmark, we identified 3585 patients with biochemically confirmed pHPT among whom *n* = 1977 (55%) were treated with PTX (PTX-group) whereas *n* = 1608 (45%) were followed without surgery (non-PTX group). Baseline was defined as time of diagnosis and kidney function was re-assessed 9–15 months after PTX (PTX group) or 9–15 months after diagnosis (non-PTX group).

**Results:**

At follow-up, eGFR had decreased significantly in the PTX- compared to the non-PTX-group (median − 4% vs. − 1%, *p* < 0.01). Stratification by baseline eGFR showed that the decrease was significant for those with a baseline eGFR value of 80–89 and > 90 mL/min, but not for those with lower eGFR values. Findings did not differ between patients with mild compared to moderate/severe hypercalcemia. However, after mutual adjustments, we identified baseline levels of calcium, PTH, and eGFR as well as age and treatment (PTX vs. no-PTX) as independent predictors for changes in kidney function.

**Conclusion:**

Compared to non-surgical surveillance, PTX is associated with a small but significant decrease in kidney function in pHPT patients with an initial normal kidney function.

## Background

Primary hyperparathyroidism (pHPT) is an endocrine disease caused by parathyroid hormone (PTH) producing adenoma or hyperplasia in one or more of the 4 parathyroid glands [1]. PTH levels are augmented due to decreased responsiveness of the glands to the normal negative feedback system via calcium-sensing receptors. The increased PTH levels cause hypercalcemia by increased skeletal calcium release, enhanced kidney tubular calcium reabsorption, and increased intestinal calcium absorption through PTH stimulated 1α-hydroxylation of vitamin D [1]. Therefore, pHPT is defined biochemically by hypercalcemia and elevated or inappropriately normal levels of PTH [1].

Parathyroidectomy (PTX) is the only curative treatment for pHPT [1, 2]. If successful, PTH and calcium levels are normalized. Some patients are followed without PTX if they do not consent to surgery or have significant operative contraindications. Moreover, if plasma calcium levels are only mildly elevated it remains an open question whether patients benefit from surgery, although guidelines recommend PTX if patients also suffer from e.g., osteoporosis or kidney diseases [3, 4].

In several studies, pHPT has been associated with an increased risk of kidney complications in terms of impaired kidney function and increased risk of kidney stones and nephrocalcinosis. Of importance, a decreased kidney function may by itself contribute to elevated PTH levels which may result in a circulus vitiosus. Accordingly, PTX is recommended by guidelines in case of kidney stones/nephrocalcinosis or if eGFR is below 60 mL/min [3, 5–10]. Randomized controlled trials (RCT’s) and retrospective studies with different follow-up time have shown a stable kidney function after PTX [1, 11–16]. Most endocrinologists would expect that PTX, with removal of the hypercalciuric drive, would lead to some improvement in kidney function over conservative treatment. However, two recent studies suggested that surgical cure of pHPT is associated with an impaired kidney function in terms of a significantly decreased eGFR after PTX in patients with pre-operative eGFR > 60 mL/min [5, 17]. The two studies followed relatively small study groups (*n* < 300) and included only patients undergoing surgery (PTX) without studding changes in groups of patients not undergoing PTX. Accordingly, no studies have so far addressed whether a similar decrease in kidney function may occur during observation *without* surgery.

Within a large cohort of patients with biochemically confirmed pHPT, we aimed to compare effects of PTX versus observation on kidney function measured after 1 year (9–15 months) of follow-up. Additionally, we stratified the results for baseline levels of kidney function as well as degree of hypercalcemia.

## Methods

### Study design

In this observational study, we retrospectively identified patients with biochemically confirmed pHPT who had been treated by PTX (PTX group) or followed without surgery (non-PTX group).

### Identification of patients

**Fig. 1:**
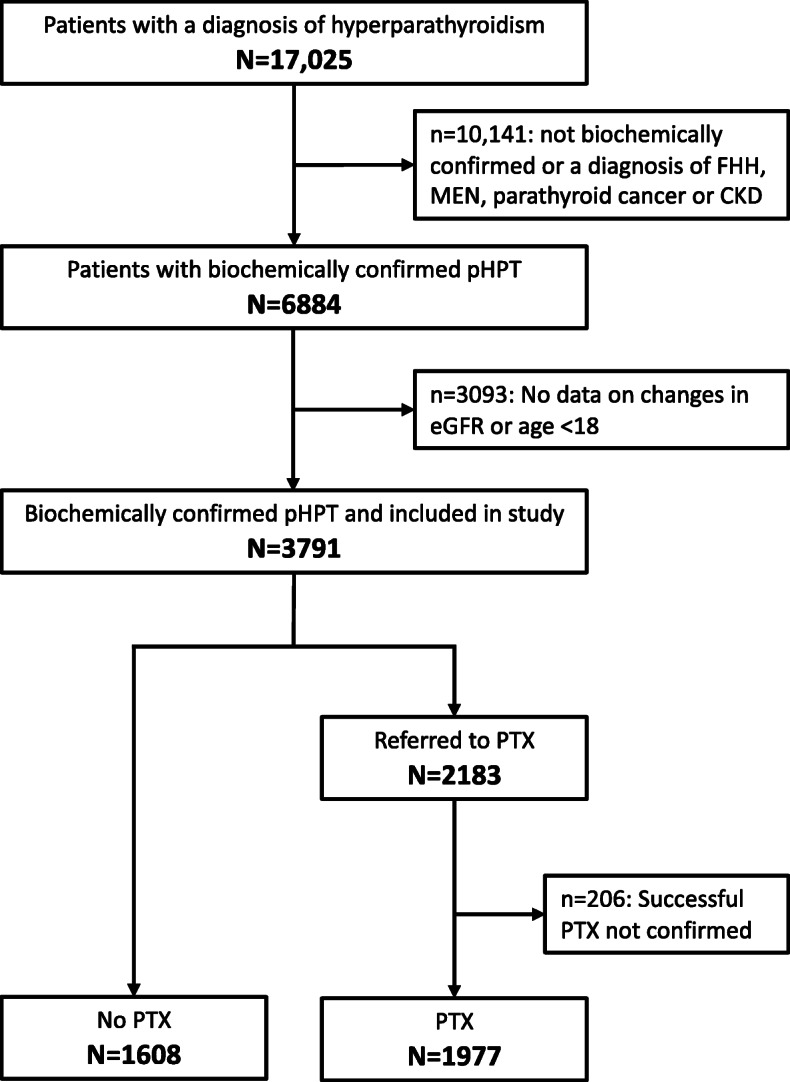
Flowchart presenting selection of the study population with primary hyperparathyroidism (pHPT). Abbreviations: FHH: familial hypocalciuric hypercalcemia; MEN: multiple endocrine neoplasia, CKD: chronic kidney disease; eGFR: estimated glomerular filtration rate; PTX: parathyroidectomy

A flow-chart presenting selection of the study population is shown in Fig. 1. In Denmark, the Danish National Patient Registry (NPR) has information on all inpatient hospital contacts since 1977. In 1995, information on outpatient contacts were added to the registry [18]. Using the NPR, we identified all patients assigned a hospital discharge code of hyperparathyroidism according to the International Classification of Diseases (ICD) between 1977 and June 2015. We searched for ICD codes according to the ICD-8th version (codes: 25200 to 25,209) which were used in Denmark from 1971 to 1993 and the 10th version (codes E21.*) which has been used since 1994. ICD version 9 was never used in Denmark. We identified a total of 17,025 patients with a diagnosis of hyperparathyroidism. Within this group of patients, we searched for biochemistry, which could confirm a diagnosis of pHPT.

The Danish Data Protection Agency was notified about the database (# 1–16–02-135-15), and the Danish Health and Medicines Authority granted access to retrieve data from hospital charts (# 3–3013-948/1).

### Biochemistry

All Danish citizens are assigned a Civil Personal Registration (CPR) number allowing linkage between different registers. Using the CPR number, we requested results of measurements of plasma PTH, calcium and creatinine levels from major Danish medical biochemistry laboratories.

We considered a diagnosis of pHPT as confirmed if blood tests on two separate occasions showed an average PTH level in the upper third- or above the upper limit of the reference interval with concomitant hypercalcemia (hyperparathyroid hypercalcemia). Since calcium levels were measured as a mixture of both ionizing and total levels at different laboratories, we accepted a diagnosis of hypercalcemia if:

- A mean of two measurements of ionized or total calcium levels were above the upper limit of the reference interval.
- An ionized and total calcium level were both above the upper limit of the reference interval, if only one measurement of ionized or total calcium levels was available.
- Hypercalcemia and a high PTH level, which were normalised following PTX, if only one measurement of plasma calcium was available.

We used reference ranges from the local laboratories performing the analyses.

### Exclusion of patients

In addition to excluding patients without a diagnosis of pHPT which could be confirmed by biochemistry, we restricted the population to only patient with sporadic pHPT by excluding patients who (ever) had been assigned a diagnosis of familial hypocalciuric hypercalcemia (FHH), multiple endocrine neoplasia (MEN) or parathyroid cancer. Furthermore, we excluded patients with kidney insufficiency diagnosed more than 1 year prior to the diagnosis of pHPT. From these criteria, 6884 patients with biochemically confirmed pHPT were identified.

As we aimed to study effects of PTX on kidney function from baseline to follow-up, we excluded patients without available measurements of plasma creatinine levels at baseline (i.e., closest to the day of diagnosis) and/or after 9–15 months of follow-up (i.e., 1 year after baseline for the non-PTX group or 1 year after PTX, respectively) (*n* = 3088). Furthermore, we excluded patients below 18 years of age (*n* = 5), as the Chronic Kidney Disease Epidemiology Collaboration (CKD-EPI) Equation used to calculate eGFR values only is considered valid for patients above 18 years of age [19]. Using this equation, we were able to account for age, sex and levels of creatinine when calculating eGFR values. Due to lack of information, we were not able to account for race. However, the majority of the Danish population are Caucasians and doubts have been raised as to whether race has a significant impact on the CKD-EPI calculation [20]. Accordingly, we identified 3791 patients with biochemically confirmed sporadic pHPT above the age of 18 years with measurements of plasma creatinine levels at baseline and follow-up.

### Identification of patients who underwent PTX

We used the Danish NPR to identify patients within this group (*n* = 3791), who had been assigned a procedural ICD-code of parathyroid or thyroid surgery (ICD-8: 080xx-085xx; ICD-10: KBAxx-KBBxx). We included a search for thyroid surgery as it is our experience that some patients with pHPT have thyroid surgery in combination with PTX, but not always are assigned an ICD-code for both procedures. Furthermore, in order to assure correct classification (PTX vs. no surgery) of patients diagnosed with pHPT just prior to end of our initial search in June 2015, we updated our search in November 2017. Thus, we identified 1608 patients who had not been assigned a procedural code as stated above, whereas 2183 patients had been treated with PTX. As we aimed to study effects of PTX on kidney function, we excluded patients with (a potential) unsuccessful PTX (*n* = 206) defined as lack of normalization of plasma calcium levels or no measurements available after surgery. Accordingly, the total study population consisted of 1977 in the intervention group (PTX-treated) and 1608 in the control (non-PTX) group).

### Statistics

We used non-parametric statistic, as a Gaussian distribution could not be confirmed for the variables. Group characteristics are reported using median with interquartile range (IQR) (25 and 75% percentiles). Group differences were assessed using Chi-square test for categorical variables. For continuous variables, we compared differences between groups using Mann-Whitney U test. For changes within the group, we tested for statistical significance using Wilcoxon Signed Rank Test.

The difference between baseline and follow-up levels of creatinine and eGFR was calculated in percentage to minimize differences caused by the varying measuring methods in the laboratories. Between groups differences in follow-up time, were accounted for by calculating average changes per year.

In sub-analyses we stratified findings according to baseline levels of kidney function and hypercalcemia.

Results were adjusted for age, sex and follow-up time in a General Linear Model using a univariate procedure. Furthermore, a multiple regression analysis was performed to assign predictors of change in kidney function.

All statistical tests were two-sided and *p*-values < 0.05 were considered statistically significant.

All calculations in the data analysis were performed using the IBM Statistical Package for Social Science (SPSS 26.0) for Mac (IBM Corp, Armonk, NY, USA).

## Results

### Baseline characteristics

Baseline characteristics are displayed in Table 1. We included a total of 3585 patients (79% females) with biochemically confirmed pHPT. At baseline, eGFR was above 90 mL/min in *n* = 964 (26.9%), between 60 to 90 mL/min in *n* = 1681 (46.9%) and below 60 mL/min in *n* = 940 (26.2%) of the patients. A total of *n* = 1977 (55%) were treated with (successful) PTX, whereas *n* = 1608 (45%) were followed without surgery. Characteristics of the non-PTX- vs PTX-group were significantly different in all studied aspects. The PTX group was younger, had higher men to women ratio and baseline biochemistry showed a better kidney function, and higher PTH and calcium levels.

**Table 1:** Characteristics study population – non-PTX vs PTX

		All	non-PTX	PTX	p
*Normal range*		(*n* = 3585)	(*n* = 1608)	(*n* = 1977)	
**Sex, n (%)**					0.024*
**Male**		752 (21%)	310 (19%)	442 (22%)	
**Female**		2833 (79%)	1298 (81%)	1535 (78%)	
**Age at diagnosis, years**		67 (57;76)	74 (64;81)	62 (54;70)	< 0.001*
**Time difference from diagnosis to follow-up, days**		433 (364;594)	365 (337;386)	575 (480;734)	< 0.001*
**Biochemistry Baseline**					
**P-Creatinin, μmol/L**	*45–90*	75 (64;91)	79 (65;98)	73 (63;87)	< 0.001*
**P-Ca (ionized), mmol/L**	*1.18–1.32*	1.43 (1.38;1.49)	1.40 (1.36;1.44)	1.45 (1.40;1.53)	< 0.001*
**P-Ca (total), mmol/L**	*2.20–2.55*	2.67 (2.58;2.78)	2.62 (2.54;2.71)	2.72 (2.63;2.84)	< 0.001*
**P-PTH, pmol/L**	*1.6–6.9*	10.90 (8.29;15;36)	9.80 (7.71;13.45)	11.85 (9.00;16.90)	< 0.001*
**eGFR, mL/min**	*> 90*	76 (59;91)	69 (52;86)	81 (66;95)	< 0.001*
**Biochemistry Follow-up**					
**P-Creatinin, μmol/L**	*45–90*	77 (65;93)	79 (65;98)	76 (65;90)	< 0.001*
**P-Ca (ionized), mmol/L**	*1.18–1.32*	1.36 (1.28;1.42)	1.38 (1.33;1.43)	1.23 (1.19;1.25	< 0.001*
**P-Ca (total), mmol/L**	*2.20–2.55*	2.59 (2.47;2.70)	2.61 (2.52;2.71)	2.32 (2.23;2.39)	< 0.001*
**P-PTH, pmol/L**	*1.6–6.9*	6.5 (4.29;9.90)	9.59 (6.90;13.52)	4.80 (3.44;6.51)	< 0.001*
**eGFR, mL/min**	*> 90*	75 (57;90)	69 (51;86)	79 (63;92)	< 0.001*

Results listed as number of subjects (%) or median with interquartile range (25%;75%)

*p*-values shows results of statistical tests comparing PTX-treated and non-PTX-treated. *p* < 0.05 is considered as significant and is marked by *

Baseline: Time of pHPT-diagnosis

Follow-up: 9–15 months after respectively time of diagnosis for non-PTX group and PTX for PTX group

*Abbreviations*: *PTX* parathyroidectomy, *pHPT* primary hyperparathyroidism, *Ca* calcium, *PTH* parathyroid hormone, *eGFR* estimated glomerular filtration rate

At follow-up, all biochemical variables were within the normal range in the PTX-group. The follow-up time was significantly longer in the PTX group. This was due to the definition of the follow-up period in the PTX group as 9–15 months after PTX, which resulted in a mean time gap of 7 months from time of diagnosis to PTX.

### Baseline to follow-up

**Fig. 2:**
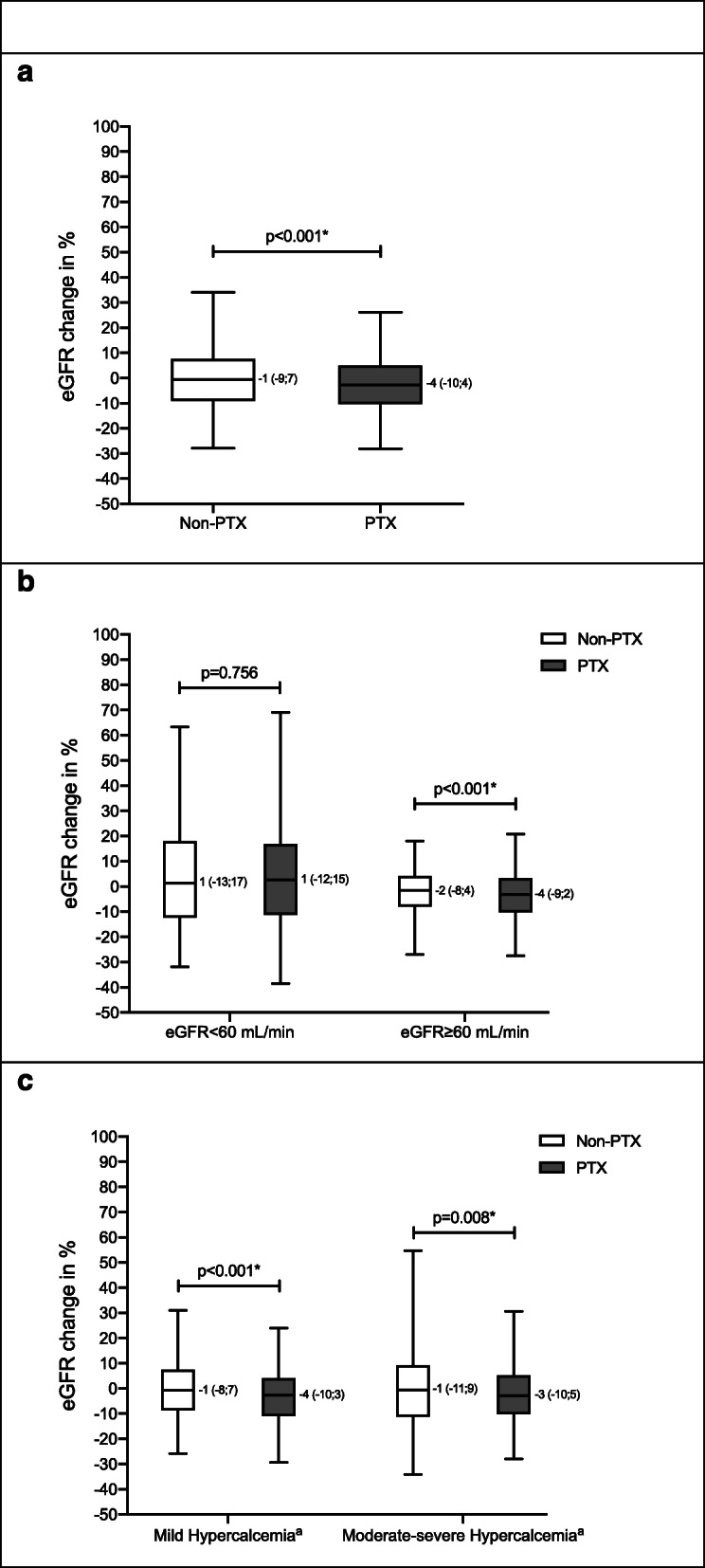
Box and whisker plots showing percentage eGFR change from baseline to follow-up for all subjects (**a**) and stratified according to level of eGFR (**b**) and hypercalcemia (**c**). Upper and lower part of the box represents interquartile range (25,75%) with median shown as the line within the box and whiskers shows 5 and 95% percentiles. PTX-treated had a statistically significant higher percentage eGFR change compared to non-PTX treated (* = *p* < 0.05). Abbreviations: eGFR: estimated glomerular filtration rate; PTX: parathyroidectomy. ^a^Footnote: Mild hypercalcemia is defined as plasma levels of ionized calcium above upper limit of normal but below 1.45 mmol/L. Moderate to severe hypercalcemia is defined as ionized calcium levels ≥1.45 mmol/L.

Compared with no surgical intervention, PTX was associated with an overall significant decrease in eGFR values (Table 2). As shown in Fig. 2a, eGFR decreased by a median of 4% in those treated with PTX whereas eGFR decreased by only 1% in the non-PTX group (*p* < 0.001). This was not changed by accounting for differences in average follow-up time i.e., eGFR decreased by a median of 2.7% per year in the PTX group and by only 0.7% per year in the non-PTX group (*p* < 0.001).

**Table 2:** Baseline to follow-up

	Non-PTX		PTX		
	Baseline	Follow-up	Baseline	Follow-up	p
**All**	(*n* = 1608)		(*n* = 1977)		
**P-Creatinine, μmol/L**	79 (65;98)	79 (65;98) *	73 (63;87)	76 (65;90) *	< 0.001
**eGFR, mL/min**	69 (52;86)	69 (51;86) *	81 (66;95)	79 (63;93) *	< 0.001
**eGFR < 60 mL/min**	(*n* = 593)		(*n* = 344)		
**P-Creatinine, μmol/L**	105 (90;127)	105 (88;126)	105 (91;129)	105 (88;130)	0.906
**eGFR, mL/min**	46 (36;54)	46 (37;56) *	51 (41;56)	50 (39;58) *	0.756
**eGFR ≥ 60 mL/min**	(*n* = 1015)		(*n* = 1633)		
**P-Creatinine, μmol/L**	69 (60;77)	69 (60;80) *	70 (61;79)	72 (63;83) *	0.002
**eGFR, mL/min**	82 (71;92)	82 (70;92) *	86 (74;97)	84 (72;95) *	< 0.001
**Mild Hypercalcemia**^a^	(*n* = 1159)		(*n* = 890)		
**P-Creatinine, μmol/L**	77 (64;96)	78 (64;97) *	72 (62;85)	74 (64;88) *	0.001
**eGFR, mL/min**	72 (53;88)	72 (53;88) *	82 (67;95)	79 (65;92) *	< 0.001
**Moderate-severe Hypercalcemia**^a^	(*n* = 342)		(*n* = 936)		
**P-Creatinine, μmol/L**	84 (67;104)	83 (66;106)	74 (63;90)	77 (65;92) *	0.002
**eGFR, mL/min**	64 (47;80)	63 (46;81)	81 (64;95)	77 (62;92) *	0.008

Results listed as median with interquartile range (25%;75%)

*p*-values shows results of statistical tests comparing PTX-treated and non-PTX-treated, regarding the percentage difference from baseline to follow-up. *p* < 0.05 is considered as significant

A significant within group change from baseline to follow-up is marked by *

Baseline: Time of diagnosis

Follow-up: 9–15 months after respectively time of diagnosis for non-PTX group and PTX for PTX group

*Abbreviations*: *PTX* parathyroidectomy, *eGFR* estimated glomerular filtration rate

^a^Mild hypercalcemia is defined as plasma levels of ionized calcium above upper limit of normal but below 1.45 mmol/L. Moderate to severe hypercalcemia is defined as ionized calcium levels ≥1.45 mmol/L. Due to missing values of ionized calcium levels at baseline, number of subjects are slightly below the number stated in Fig. 1

Stratification by kidney function at baseline showed that the decline in kidney function following PTX was only significant in the group of patients with a baseline eGFR > 60 mL/min, whereas eGFR did not change significantly compared to the non-PTX group if baseline eGFR was < 60 mL/min (Fig. 2b). Analysing changes in plasma creatinine levels showed similar findings (Table 2). Results were not changed by adjustment for age and sex (data not shown).

Further stratification of baseline eGFR values into eight categories (< 30, 30–39, 40–49, 50–59, 60–69, 70–79, 80–89, and > 90 mL/min) showed that only patients with a baseline eGFR between 80 and 89 mL/min and > 90 mL/min had a significant decline in kidney function following PTX compared to the non-PTX group (Table 3).

**Table 3:** eGFR change in %, stratified by baseline eGFR

	Non-PTX	PTX	p
**eGFR < 30 mL/min**	*n* = 71	*n* = 33	
**∆ eGFR in %**	21 (−13;59)	27 (−17;81)	0.685
**eGFR 30–40 mL/min**	*n* = 126	*n* = 46	
**∆ eGFR in %**	3 (−12;19)	5 (−14;17)	0.993
**eGFR 40–50 mL/min**	*n* = 165	*n* = 84	
**∆ eGFR in %**	1 (−11;16)	5 (−11;19)	0.381
**eGFR 50–60 mL/min**	*n* = 231	*n* = 181	
**∆ eGFR in %**	0 (−12;10)	0 (−9;13)	0.811
**eGFR 60–70 mL/min**	*n* = 227	*n* = 266	
**∆ eGFR in %**	0 (−9;11)	-1 (− 9;12)	0.507
**eGFR 70–80 mL/min**	*n* = 235	*n* = 343	
**∆ eGFR in %**	−2 (−12;2)	−2 (−9;8)	0.389
**eGFR 80–90 mL/min**	*n* = 263	*n* = 350	
**∆ eGFR in %**	−2 (−9;3)	−4 (−13;3)	0.025*
**eGFR > 90 mL/min**	*n* = 290	*n* = 674	
**∆ eGFR in %**	−2 (−6;1)	−4 (−10;1)	0.001*

Results are listed as median with interquartile range (25%;75%)

*p*-values shows results of statistical tests comparing PTX-treated and non-PTX-treated. *p* < 0.05 is considered as significant and is marked by *

*Abbreviations*: *PTX* parathyroidectomy, *eGFR* estimated glomerular filtration rate

Stratification by the degree of hypercalcemia showed similar findings in the group of patients with only mild hypercalcemia (ionized calcium < 1.45 mmol/L) as in those with moderate to severe hypercalcemia (Fig. 2c and Table 2).

### Predictors of change in kidney function

Table 4 shows the results of the multiple regression analyses on potential predictors for the percentage change of eGFR. Included variables were the baseline characteristics found to differ statistically significantly between the PTX- and non-PTX-group. Total-calcium was not included in the model, because it is interrelated with levels of ionized calcium, same as for eGFR at baseline which is interrelated with age and creatinine at baseline. After mutual adjustments, PTX was associated with a decrease in eGFR. Moreover, the percentage change in eGFR was inversely associated with age and PTH levels, whereas creatinine levels at baseline and plasma ionized calcium levels at baseline were positively associated with percentage change in eGFR. Furthermore, male sex was associated with a decrease in eGFR.

**Table 4:** Multiple regression analyses

	Standardized coefficient	
	Beta	p
Sex, (female = 0/male = 1)	− 0.131	< 0.001
Age at baseline, years	−0.102	< 0.001
Plasma Creatinine at baseline, μmol/L	0.508	< 0.001
Plasma Ca^2+^ at baseline, mmol/L	0.185	< 0.001
Plasma PTH at baseline, pmol/L	−0.220	< 0.001
Parathyroidectomy, (no = 0/yes = 1)	−0.075	< 0.001

Multiple regression analyses on predictors of percentage change in eGFR after 1 year (9–15 months) follow-up in patients with pHPT

*Abbreviations*: *eGFR* estimated glomerular filtration rate, *pHPT* primary hyperparathyroidism, *Ca* calcium, *PTH* parathyroid hormone

## Discussion

Results from our historic cohort study showed that after 9–15 months follow-up, surgical cure of pHPT is associated with a decline in kidney function compared to surveillance without surgical intervention. Subgroup analyses showed that this especially applies to those with an initial (near-) normal kidney function, whereas no decline in eGFR was observed if kidney function was impaired at baseline.

Our results are in accordance with the findings from two previous retrospectives studies by Tassone et al. [5] and Garcia-Martin et al. [17]. In both studies, kidney function was assessed prior to and following PTX. Our study provides further support for these findings by including a group of patients not treated by PTX, allowing for comparisons between PTX and non-PTX treated patients. Furthermore, our findings extend previous findings by including a much larger sample of patients, allowing for a more detailed assessment of the importance of kidney function at baseline. Whereas previous studies showed that PTX is associated with a decline in kidney function only if eGFR at baseline is above 60 mL/min, but not if eGFR at baseline is below 60 mL/min, our analyses suggest that the decline in kidney function following PTX only applies to those with a baseline eGFR above 80 mL/min.

PHPT is associated with an increased risk of kidney impairment through several of different mechanisms which include hypercalciuria, development of kidney calcifications and higher rate of hypertension [21, 22].

Similar to previous studies, our study showed a high prevalence of kidney impairment as 26.2% of our included patients had an eGFR below 60 mL/min at baseline [23–25]. However, during 1 year of surveillance without surgery, eGFR remained stable in the non-PTX group. This is consistent with several previous studies showing that kidney function remains largely stable during different follow-up times [11, 12, 26, 27].

Thus, no rapid decline in kidney function occurs shortly after diagnosis in patients managed conservatively. pHPT is known as a disease that often remains silent for several years before diagnosed [28]. Several of the patients included in our study had presumably been suffering from pHPT for a long period prior to diagnosis, which may have resulted in kidney impairment. Of importance, the decline in kidney function following PTX was only of small magnitude in absolute terms and probably without clinical importance. Accordingly, despite a small decrease in eGFR following PTX, it seems appropriate to shorten the duration of the disease by surgical cure and thereby presumably lower the risk of further (long-term) kidney complications. Moreover, successful PTX is associated with additional beneficial effects such as improved BMD with reduced risk of fracture and a better quality of life [27, 29–36].

The mechanism causing a decreased eGFR following PTX needs to be further elucidated. It may be speculated whether it is due to the well-known effect of PTH as a vasodilator [37] or the diuretic actions of hypercalciuria. Thus, activation by calcium of the ductal apical calcium-sensing receptor may inhibit arginine vasopressin (AVP) mediated increases in intracellular cAMP and increase water excretion [38]. Concerning PTH as a vasodilator, a successful PTX could result in relative kidney vasoconstriction, followed by a decrease in eGFR. A similar effect might also be attributable to the lowering of aldosterone levels in response to PTX. PHPT has been suggested to increase plasma levels of aldosterone, with a reduction following PTX [39, 40]. The reduced aldosterone levels will lower fluid volume and thereby increase plasma creatinine levels and decrease eGFR. This mechanism has been well described following adrenalectomy in patients treated for primary hyperaldosteronism [41, 42]. Moreover, muscle function is known to be impaired in patients with pHPT. Muscle function may increase following cure for pHPT which may contribute to increased levels of creatinine [43].

Of interest, in our mutually adjusted multiple regression analyses, PTH levels at baseline were inversely associated with percentage change in eGFR. As no effect was observed in patients with impaired kidney function, it may be speculated whether PTH only causes kidney vasodilation if kidney function is well preserved. Of interest, PTH therapy of hypoparathyroidism for 8 years has been associated with a stable kidney function, whereas a decline in kidney function often occurs if patients are treated with conventional therapy in terms of active vitamin D and calcium supplements [44]. Further studies should aim to investigate if this potential renoprotective effect of PTH replacement therapy in hypoparathyroidism only applies to patients with an initial normal kidney function or also may be beneficial to patients with impaired kidney function. Even if endogenous PTH acts as a mild kidney vasodilator in pHPT, any protective effects would come at the cost of an increased risk of kidney calculi and it is important to stress that patients in conservative treatment would be monitored for the development of stone disease or nephrocalcinosis and that developing this complication would be a strong indication for fast-tracking them for parathyroidectomy.

### Strength and limitations

The strength of this study is firstly the large cohort which includes both a group that has received PTX and a control group, opposite the recent studies only investigating kidney function prior to and after PTX. However, our study design using a historic cohort makes it impossible to draw causal conclusions from our findings. As shown in Table 1, characteristics between PTX-treated and non-PTX treated patients differ on several parameters. Certainly, conservatively managed patients were older, had lower serum calcium levels and poorer kidney function at baseline. There is some obvious selection bias because the groups are not randomized, and there are reasons causing patients to be operated or not. Guidelines strongly favour surgery in patients with impaired kidney function, but young age is an additional factor than strongly speaks for PTX in endocrine practice. In addition to the question of surgery referral bias, we do not have information on whether patients have received medical treatment (e.g., bisphosphonates, cinacalcet etc.). Such medical treatment could influence kidney function. Although kidney function is known to decrease with age, we do not believe that the longer follow-up time in the PTX compared to the non-PTX group can explain the significantly more pronounced decrease in e-GFR in the PTX group, as the difference in follow-up time was only 7 months. Furthermore, analyses adjusted for difference in follow-up time did not change the result.

Another strength of our study is that our data is based on the Danish NPR, which is a well-validated database [18]. Data on biochemistry were available from 1977, the year when NPR was founded. It was only possible to validate the pHPT diagnosis by biochemistry in 1/3 of the patients. Of importance, our initial search for patients in the Danish NPR was very broad in order to aim at identifying as many patients with pHPT as possible. Many of the patients initially identified, were suffering from other causes of hyperparathyroidism. Thus, the fact that many of the patients initially identified could not be confirmed as suffering from pHPT should not be interpreted as a lack of specificity of the register but as a consequence of a broad search strategy with deliberately high sensitivity. Furthermore, we were only able to search for biochemical findings in databases from the major current laboratories. As our study includes a time span of more than 40 years, some laboratories do no longer exist or have not maintained their database on biochemical findings for the entire time period. On the other hand, we feel very confident that patients identified were suffering from pHPT. There is no reason to assume a connection between, whether biochemistry was available for a patient and the change in kidney function after PTX/non-PTX-treatment, why we do not think that this is a cause of selection bias. Furthermore, follow-up after 9–15 months was only registered in half of the patients with a confirmed diagnosis. This is probably due to the retrospective design of this study, where patients have been followed in the clinic and not according to a predefined protocol.

Biochemistry was performed by various laboratories and we have no information on the exact measurement methods. We tried to compensate for this by using local reference ranges used by the specific laboratories in the relevant periods. In addition, to minimize differences caused by the different measurement methods, a percentage difference between baseline and follow-up levels of creatinine and eGFR was calculated, as most patients had these measurements performed in the same laboratory.

Comparing our study with similar studies, other studies used additional exclusion criteria for the study population, like in the study from Tassone et al., thyroid and liver disease patients were excluded [5]. We did not include information on comorbidities, although this could have added information on studied patients.

Furthermore, it would have been of advantage if aldosterone levels had been included to test the hypothesis of an effect on eGFR of lowering aldosterone levels in response to PTX. However, aldosterone levels are not measured on a regular basis in patients with PHPT, and therefore aldosterone levels are not captured in our database. Nevertheless, we excluded patients with known kidney impairment > 1 year before the time of diagnosis, to eliminate other reasons of kidney impairment as well as tertiary hyperparathyroidism as a reason for the hyperparathyroid hypercalcemia.

We cannot exclude that the observed effect on kidney function may be due to regression towards the mean. Prospective studies are needed to get a more profound understanding of this. Lastly, as pHPT is considered a slowly progressing disease, our follow-up time (9–15 months) was relatively short. In further studies, it could be of interest to study the long-term effects of PTX on kidney function.

## Conclusion

In conclusion, our study showed that PTX is associated with a clinically small but statistically significant decrease in kidney function after 9–15 months follow-up compared to pHPT patients followed without surgery. As it apparently only applies to patients with an initial (near-) normal kidney function, we do not think that this should be of major concern when deciding whether a patient with pHPT should be referred to PTX. Our findings are of interest in terms of the physiological effects of PTH on kidney function, which should be addressed more closely in further studies. Our findings do not exclude the possibility that long term kidney outcomes could be more favourable with PTX than with conservative treatment, but we did not find it to be the case in the short term.

## Data Availability

Data supporting the findings of this study are available from the corresponding author upon reasonable request.

## Abbreviations

BMD: Bone mineral density
eGFR: Estimated glomerular filtration rate
FHH: Familial hypocalciuric hypercalcemia
HPT: Hyperparathyroidism
MEN: Multiple endocrine neoplasia
NPR: National Hospital Patient Registry
pHPT: Primary hyperparathyroidism
PTH: Parathyroid hormone
PTX: Parathyroidectomy
